# Switching to High-Dose Aflibercept (8 mg) with Pro Re Nata Reduces Treatment Burden in Diabetic Macular Edema: A Real-World Pilot Study

**DOI:** 10.3390/jcm15062210

**Published:** 2026-03-14

**Authors:** Masahiko Funatsu, Fumiaki Higashijima, Nobuaki Ariyoshi, Aiko Haraguchi, Yuki Wasai, Masanori Mikuni, Manami Ohta, Makiko Wakuta, Shinji Hirano, Kazuhiko Yamauchi, Kazuhiro Kimura

**Affiliations:** 1 Department of Ophthalmology, Yamaguchi University Graduate School of Medicine, Ube 755-8505, Yamaguchi, Japan; orange9@yamaguchi-u.ac.jp (M.F.); higashi@yamaguchi-u.ac.jp (F.H.); mwakut@yamaguchi-u.ac.jp (M.W.);; 2 Yamaguchi Red Cross Hospital, Yamaguchi 753-0092, Yamaguchi, Japan

**Keywords:** diabetic macular edema, aflibercept 8 mg, pro re nata, PRN, treatment switching, real-world evidence, anti-VEGF therapy, injection frequency

## Abstract

**Background/Objectives:** The PHOTON trial established the efficacy of aflibercept 8 mg using fixed-interval dosing in treatment-naïve patients; however, real-world evidence regarding pro re nata (PRN) regimens in switch cases remains limited. This pilot study evaluated the short-term efficacy and safety of switching to aflibercept 8 mg with PRN dosing in eyes with DME. **Methods:** This retrospective study included 20 eyes from 12 patients with DME who switched to aflibercept 8 mg and were followed for 6 months. Patients received initial induction doses (1–3 injections based on predetermined anatomical and functional criteria) followed by PRN dosing based on clinical findings. Primary outcomes were changes in best-corrected visual acuity (BCVA) and central retinal thickness (CRT). Treatment intervals and injection frequency were also analyzed. **Results:** Mean logMAR BCVA was maintained from baseline (0.242 ± 0.252) throughout the follow-up period: 0.164 ± 0.218 at 1 month, 0.138 ± 0.241 at 2 months, 0.145 ± 0.204 at 3 months, 0.143 ± 0.181 at 4 months, 0.149 ± 0.166 at 5 months, and 0.180 ± 0.224 at 6 months. No statistically significant changes in BCVA from baseline were observed at any time point. Mean CRT decreased from baseline (369.6 ± 138.3 μm) at all follow-up time points: 251.5 ± 82.1 μm at 1 month, 269.1 ± 104.5 μm at 2 months, 255.8 ± 67.8 μm at 3 months, 275.2 ± 76.6 μm at 4 months, 301.4 ± 81.2 μm at 5 months, and 302.7 ± 86.8 μm at 6 months. Statistically significant reductions in CRT were observed at 1 through 4 months (1 month: *p* = 0.000010; 2 months: *p* = 0.000243; 3 months: *p* = 0.000035; 4 months: *p* = 0.000597), whereas the reductions at 5 months (*p* = 0.0317) and 6 months (*p* = 0.0424) were not statistically significant. The mean number of injections over 6 months was 1.45 ± 1.05 (median 1; range 1–4), with 70% of eyes achieving treatment intervals ≥ 4 months. Five eyes (25%) required only the switching dose with no additional treatment during follow-up. No intraocular inflammation or retinal vasculitis was observed. **Conclusions:** Switching to aflibercept 8 mg with PRN dosing provided sustained anatomical improvement and maintained visual acuity in DME, with one quarter of the cases maintaining these outcomes with only a single additional injection. These real-world findings from a pilot study suggest that the PRN approach appears feasible and effective in real-world practice, offering a practical treatment option that may help reduce treatment burden while maintaining disease control.

## 1. Introduction

Diabetic macular edema (DME) is a major cause of vision loss in patients with diabetic retinopathy, and vascular endothelial growth factor (VEGF) plays a central role in its pathogenesis [1]. Intravitreal anti-VEGF therapy has become the established first-line treatment for DME, with the efficacy of aflibercept 2 mg well documented in pivotal trials such as VIVID and VISTA [2,3].

The recent introduction of aflibercept 8 mg represents a significant advancement in anti-VEGF therapy. This high-dose formulation contains a fourfold increase in drug concentration compared with aflibercept 2 mg, offering enhanced VEGF-neutralizing capacity and the potential for extended treatment intervals [4,5]. The PHOTON trial demonstrated that aflibercept 8 mg achieved non-inferior visual acuity gains compared to aflibercept 2 mg, with dosing intervals of 12 or 16 weeks following three initial monthly doses [6]. At 96 weeks, 89% of patients maintained ≥12-week dosing intervals, and 27% achieved 24-week intervals [7].

However, an important limitation of the PHOTON trial was its use of fixed-interval dosing with predefined dose regimen modification criteria. In real-world clinical practice, particularly in resource-constrained settings, pro re nata (PRN; as-needed) regimens are more commonly employed due to practical considerations including patient compliance, healthcare resource availability, and reimbursement constraints [8,9]. Despite the widespread use of PRN regimens with aflibercept 2 mg for DME [10,11], data on aflibercept 8 mg administered via PRN protocols remain extremely limited.

The treat-and-extend (T&E) regimen, while demonstrating superior outcomes to PRN in some studies with aflibercept 2 mg [12,13], is also disease-guided but differs from PRN in that treatment is administered at every scheduled visit while the interval between visits is adjusted according to disease activity; this approach requires strict adherence to scheduled follow-ups, which may not be feasible in all clinical environments. In contrast, PRN dosing allows treatment intervals to be adjusted based on disease activity at each visit, offering greater flexibility—yet its performance with aflibercept 8 mg in DME has not been systematically evaluated.

Furthermore, switching therapy has become increasingly common in cases with insufficient response to existing treatments. Several studies have reported that switching from aflibercept 2 mg to faricimab can improve central retinal thickness (CRT) and short-term visual outcomes in DME [14,15], but comparative data for aflibercept 8 mg switches, particularly using PRN dosing, are lacking. In our clinical practice, switching to aflibercept 8 mg was considered for eyes with insufficient anatomical response or recurrent edema despite prior anti-VEGF therapy, as well as for patients requiring frequent injections and experiencing a high treatment burden. Extending the treatment interval was also an important reason for considering the switch.

Therefore, this study aimed to retrospectively evaluate the short-term efficacy and safety of switching to aflibercept 8 mg using a PRN regimen in eyes with DME over a 6-month follow-up period. We specifically assessed changes in visual acuity, CRT, injection frequency, treatment intervals, and safety outcomes to provide real-world evidence for this clinically relevant treatment approach.

## 2. Materials and Methods

### 2.1. Study Design and Patient Selection

This retrospective, observational study reviewed medical records of consecutive patients with DME who underwent intravitreal injection therapy at Yamaguchi University Hospital and Yamaguchi Red Cross Hospital between January 2024 and July 2025. Patients were included if they: (1) had center-involving DME confirmed by optical coherence tomography (OCT); (2) switched to aflibercept 8 mg from other anti-VEGF agents or corticosteroids; (3) had a follow-up period of at least 6 months after switching; and (4) received treatment according to a PRN protocol. Exclusion criteria included active proliferative diabetic retinopathy requiring panretinal photocoagulation, uncontrolled glaucoma, significant media opacity, or concurrent other macular disease.

This study was approved by the Research Ethics Committee (REC) of Yamaguchi University Hospital and adhered to the tenets of the Declaration of Helsinki (2024 Revision). The REC waived the need for written informed consent for all subjects and approved the use of an opt-out consent method for this study.

### 2.2. Treatment Protocol

#### 2.2.1. Induction Phase

Following the switch to aflibercept 8 mg, patients received an individualized induction phase based on baseline disease activity and prior treatment history. The induction regimen consisted of 1–3 monthly injections. The number of injections was determined by the treating physician according to anatomical and functional response. Additional induction injections (i.e., the second or third loading dose) were administered when any of the following conditions were met: macular edema persisted on OCT, the CRT had not yet reached its lowest historical level, or the previous injection had resulted in visual improvement and further visual gain was expected. These criteria reflected the physician’s assessment of ongoing disease activity and the need for additional loading.

#### 2.2.2. PRN (Pro Re Nata) Maintenance Phase

After the induction phase, patients entered a PRN maintenance regimen. Monthly follow-up visits were scheduled, at which time treatment decisions were made based on clinical and anatomical criteria. Retreatment criteria included any of the following:

- Decrease in BCVA ≥ 5 letters (Early Treatment Diabetic Retinopathy Study [ETDRS] equivalent) from the previous visit attributed to DME
- Increase in CRT ≥ 50 μm from the previous visit
- Presence or worsening of intraretinal or subretinal fluid on OCT
- New or persistent macular edema involving the central fovea

Patients were not retreated if they demonstrated stable or improved vision with no signs of disease activity on OCT examination.

### 2.3. Data Collection

Baseline data collected included: age, sex, eye laterality, dialysis history, history of pars plana vitrectomy (PPV), lens status, history of photocoagulation, diabetic retinopathy severity classification, type and number of previous intravitreal injections, and treatment intervals during the 6 months prior to switching.

At baseline and each monthly visit, best-corrected visual acuity (BCVA) was measured using a standard Landolt C chart and converted to logarithm of the minimum angle of resolution (logMAR) for analysis. Central retinal thickness (CRT) was measured using swept-source OCT (DRI OCT-1 Atlantis, Topcon Corporation, 120 Tokyo, Japan).

### 2.4. Outcome Measures

The primary outcome was the change in logMAR BCVA from baseline at 1, 2, 3, 4, 5, and 6 months after the first aflibercept 8 mg injection. Secondary outcomes included: (1) change in CRT from baseline at each monthly time point; (2) the number of aflibercept 8 mg injections administered during the 6-month follow-up period; (3) mean treatment intervals between injections; (4) proportion of eyes achieving treatment intervals ≥ 4 months; and (5) incidence of ocular and systemic adverse events.

### 2.5. Statistical Analysis

Continuous variables are expressed as mean ± standard deviation (SD). Changes in BCVA and CRT from baseline at each time point were analyzed using generalized linear mixed-effects models. Missing data were minimal in this cohort, and no imputation methods, including LOCF, were applied; all analyses were performed using observed data. For the primary outcomes, multiple comparisons were corrected using the Bonferroni method, resulting in a significance threshold of *p* < 0.0083. A pre–post comparison of injection frequency was performed using the Wilcoxon signed-rank test, with statistical significance set at *p* < 0.05. Statistical analyses were conducted using EZR software version 1.61 (Saitama Medical Center, Jichi Medical University, Saitama, Japan).

## 3. Results

### 3.1. Patient and Eye Characteristics

A total of 20 eyes from 12 patients met the inclusion criteria and were analyzed. Patient demographics and baseline characteristics are summarized in Table 1. The mean age was 71.5 ± 14.0 years. Five patients (42%) were female, and two patients (17%) were receiving dialysis. The majority of eyes were pseudophakic (17 eyes, 85%), and 2 eyes (10%) had a history of pars plana vitrectomy.

Prior to switching to aflibercept 8 mg, most eyes had been treated with aflibercept 2 mg (17 eyes, 85%), while 2 eyes (10%) had received faricimab and 1 eye (5%) had been treated with intravitreal triamcinolone acetonide. During the 6 months preceding the switch, the mean injection interval was 81.16 ± 50.68 days, and the mean number of injections was 1.55 ± 1.15.

### 3.2. Visual Acuity and Anatomical Outcomes

**Figure 1:**
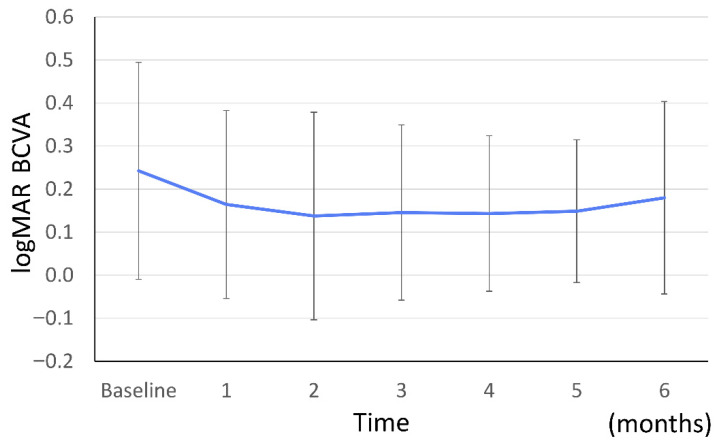
The mean logMAR BCVA changes after intravitreal aflibercept 8 mg injection. Error bars represent standard error of the mean (SEM). No statistically significant changes in BCVA from baseline were observed at any time point.

Mean logMAR BCVA was maintained from baseline (0.242 ± 0.252) throughout the follow-up period: 0.164 ± 0.218 at 1 month, 0.138 ± 0.241 at 2 months, 0.145 ± 0.204 at 3 months, 0.143 ± 0.181 at 4 months, 0.149 ± 0.166 at 5 months, and 0.180 ± 0.224 at 6 months (Figure 1). After applying Bonferroni correction for multiple comparisons, no statistically significant changes in BCVA from baseline were observed at any time point (1 month: adjusted *p* = 0.111; 2 months: adjusted *p* = 0.009; 3 months: adjusted *p* = 0.054; 4 months: adjusted *p* = 0.018; 5 months: adjusted *p* = 0.032; 6 months: adjusted *p* = 0.928). These results indicate that BCVA was maintained without significant improvement or deterioration after switching to aflibercept 8 mg.

**Figure 2:**
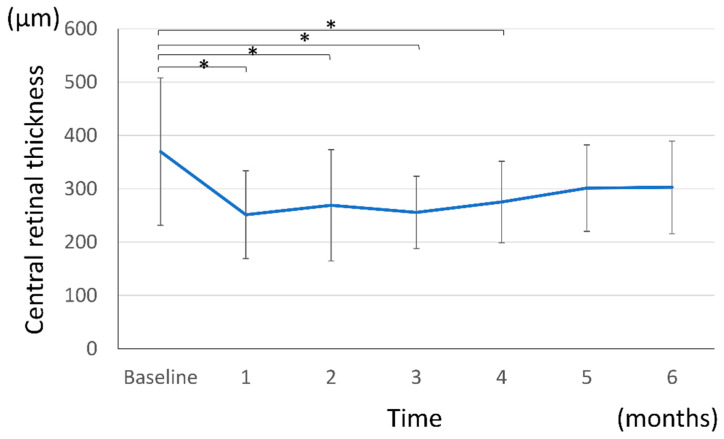
The mean retinal thickness changes after the intravitreal aflibercept 8 mg injection. Error bars represent standard error of the mean (SEM). * *p* < 0.0083 in the mixed-effects analysis with Bonferroni correction.

Mean CRT decreased from baseline (369.6 ± 138.3 μm) at all follow-up time points: 251.5 ± 82.1 μm at 1 month, 269.1 ± 104.5 μm at 2 months, 255.8 ± 67.8 μm at 3 months, 275.2 ± 76.6 μm at 4 months, 301.4 ± 81.2 μm at 5 months, and 302.7 ± 86.8 μm at 6 months (Figure 2). Mixed-effects model analysis with Bonferroni correction confirmed significant reductions in CRT at 1 through 4 months (1 month: *p* = 0.000010; 2 months: *p* = 0.000243; 3 months: *p* = 0.000035; 4 months: *p* = 0.000597), whereas the reductions at 5 months (*p* = 0.0317) and 6 months (*p* = 0.0424) were not statistically significant. The significant anatomical improvement observed during the first 4 months, followed by a gradual loss of statistical significance, may reflect partial disease reactivation in some eyes under the PRN regimen. Notably, BCVA was maintained throughout the observation period despite this trend, suggesting that the anatomical changes during the later follow-up period did not result in functional deterioration.

### 3.3. Treatment Pattern with PRN Regimen (Figure 3)

Following the switch to aflibercept 8 mg, eyes received an individualized induction phase: 14 eyes (70%) received 1 injection, 2 eyes (10%) received 2 injections, and 4 eyes (20%) received 3 consecutive monthly injections. Over the entire 6-month follow-up period, the mean number of injections per eye was 1.45 ± 1.05 (median 1; range 1–4), with a mean treatment interval of 72.37 ± 49.83 days between injections under the PRN protocol.

A pre–post comparison of injection frequency was performed using the Wilcoxon signed-rank test. The mean number of injections decreased from 1.55 before switching to 1.45 after switching, and this reduction was statistically significant (*p* = 0.00231).

Notably, 5 eyes (25%) required only the single switching injection and did not need additional treatment during the entire 6-month observation period. This may reflect favorable disease stability in some eyes; however, the possibility of selection bias should be considered when interpreting this finding.

Among the 4 eyes that received 3 consecutive doses during the induction phase, 3 eyes (75%) subsequently maintained stability without requiring any further injections for more than 4 months. Overall, 14 of 20 eyes (70%) achieved treatment intervals of ≥4 months during the follow-up period under PRN dosing.

In contrast, 2 eyes (10%) demonstrated persistent disease activity and required regular treatment every 2 months throughout the observation period despite showing anatomical improvement, suggesting factors beyond VEGF may contribute to recurrent edema in these cases.

**Figure 3:**
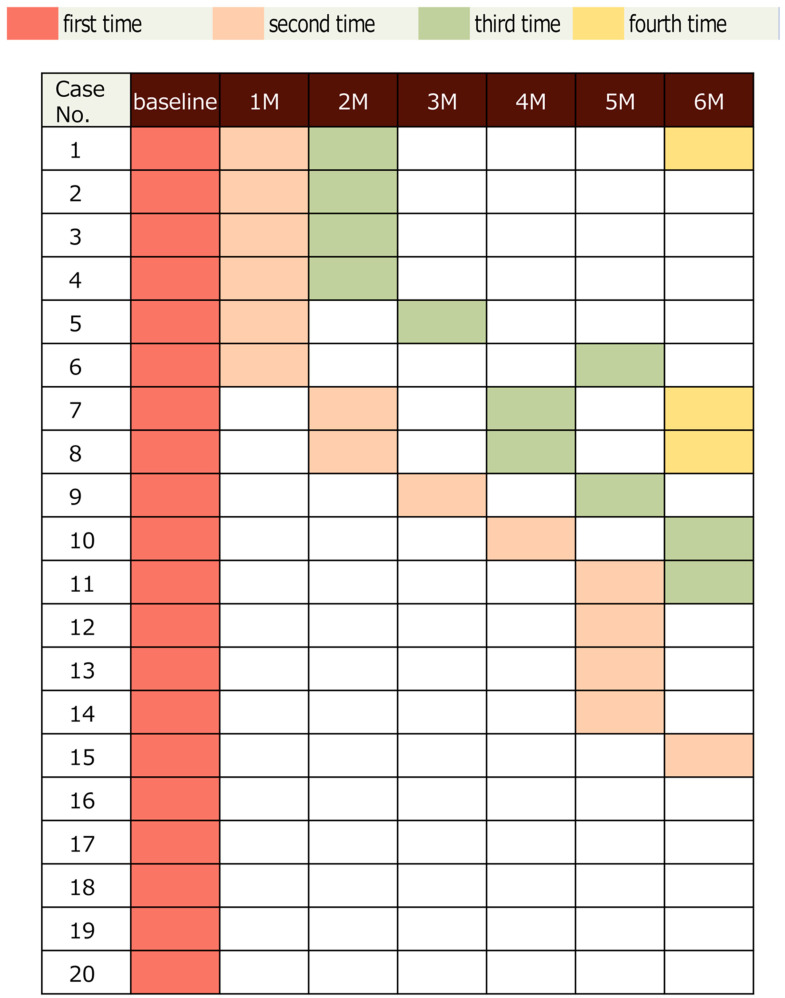
This figure shows the month and number of times aflibercept 8 mg intravitreal injections were administered after baseline in all cases. This chart color-codes the months in which intravitreal injections were administered during the 6-month period following the switch in 20 eyes. The first injection is shown in red, the second in orange, the third in green, and the fourth in yellow.

**Figure 4:**
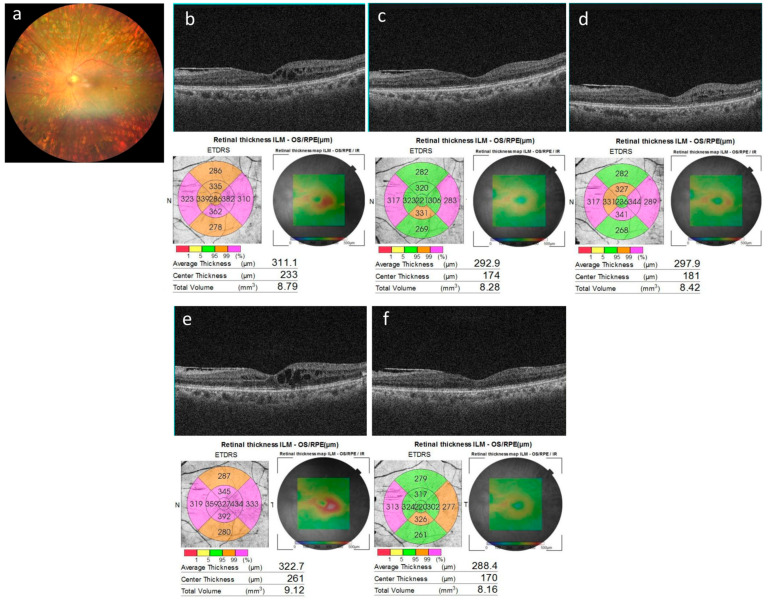
A switch case (case 14) with good response to intravitreal aflibercept 8 mg injection. (**a**) A color fundus photograph at baseline; (**b**–**f**) horizontal cross-sectional optical coherence tomography images. Macular edema observed at baseline (**b**) disappeared 1 month after the intravitreal aflibercept 8 mg injection (**c**). No recurrence of macular edema was noted for up to 4 months without additional treatment (**d**). Recurrence occurred at 5 months (**e**), but the macular edema resolved the following month after an additional intravitreal aflibercept 8 mg injection (**f**).

**Figure 5:**
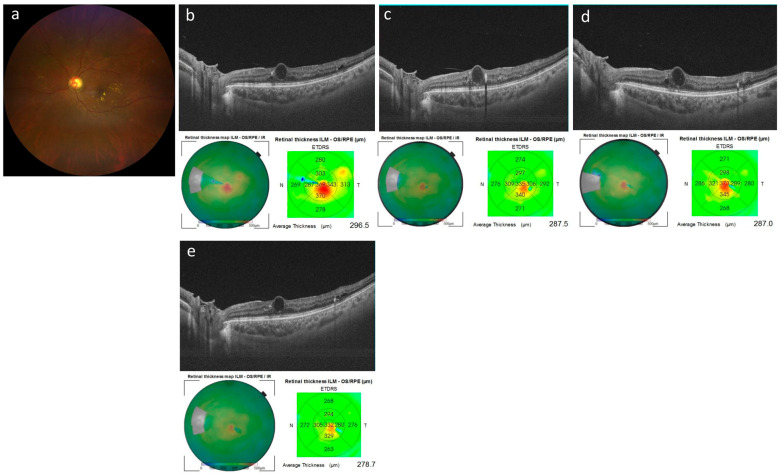
A case (case 7) showing resistance even after switching to aflibercept 8 mg injection. (**a**) Baseline color fundus photograph; (**b**–**d**) horizontal cross-sectional optical coherence tomography (OCT) images. The cystoid macular edema observed at baseline (**b**) remained unchanged after an intravitreal aflibercept 8 mg injection at 2 months (**c**). At 4 months, OCT images demonstrated refractory macular edema despite an additional intravitreal aflibercept 8 mg injection (**d**). At 6 months, OCT images demonstrated refractory macular edema despite yet another additional intravitreal aflibercept 8 mg injection (**e**).

Figure 4 shows cases that responded well after switching to aflibercept 8 mg injection, while Figure 5 shows cases that remained resistant even after switching to aflibercept 8 mg injection.

### 3.4. Safety Outcomes

No cases of endophthalmitis, retinal detachment, intraocular inflammation, or retinal vasculitis were observed during the 6-month follow-up period. No systemic thromboembolic events were reported. The safety profile of aflibercept 8 mg with PRN dosing was favorable in this DME cohort. However, given the small sample size and limited follow-up duration, these observations should be interpreted with caution, and definitive conclusions regarding safety cannot be drawn.

## 4. Discussion

This retrospective study evaluated the real-world effectiveness of aflibercept 8 mg administered via a PRN regimen in eyes with DME following a switch from other anti-VEGF agents or corticosteroids. Our findings demonstrate that switching to aflibercept 8 mg with PRN dosing provides early anatomical improvement while maintaining visual acuity while reducing injection frequency compared to the pre-switch period; although the absolute reduction was small (1.55 to 1.45 injections), this difference was statistically significant (*p* = 0.00231, Wilcoxon signed-rank test).

### 4.1. Real-World PRN Dosing: Clinical Rationale and Relationship to the PHOTON Trial

The pivotal PHOTON trial established the efficacy of aflibercept 8 mg using fixed dosing intervals of 12 or 16 weeks (following 3 initial monthly doses) with protocol-specified dose-modification criteria [6,7]. While PHOTON demonstrated impressive visual outcomes, the fixed-interval approach may not reflect real-world practice in many healthcare settings where PRN regimens predominate [8,9].

Our study addresses this important gap by evaluating aflibercept 8 mg using a flexible PRN protocol, which allows treatment intervals to be determined by disease activity rather than predetermined schedules. In the current study, eyes received a mean of only 1.45 injections over 6 months, with 25% requiring only the switching dose.

It should be noted, however, that direct numerical comparison with PHOTON is not appropriate given the fundamental differences in patient population (treatment-naïve vs. previously treated), treatment regimen (fixed-interval vs. PRN), and overall study design. PHOTON data are therefore referenced solely as contextual background. Our findings indicate that PRN dosing with aflibercept 8 mg may help reduce treatment burden in appropriately selected eyes.

### 4.2. PRN Dosing: Clinical Advantages and Practical Considerations

PRN dosing offers several practical advantages in real-world clinical practice. First, it reduces the burden of unnecessary clinic visits and injections for eyes with excellent disease control, as exemplified by the 25% of eyes requiring only the switching injection over 6 months. Second, PRN protocols allow for individualized treatment intensification in eyes with more active disease. Third, PRN dosing is often preferred by patients and may improve long-term adherence [16].

In our cohort, vision and anatomical outcomes were generally maintained over the 6-month follow-up period. In this study, the injection interval—rather than the monitoring interval—was extended, as patients continued to be monitored monthly. The BCVA showed no statistically significant change at any time point, consistent with maintenance of visual function, consistent with the limited functional gains often seen in switch populations. Although the mean BCVA improvement was modest (~0.08–0.09 logMAR), this pattern aligns with the clinical characteristics of chronic or previously treated DME. Long-standing edema can lead to irreversible outer retinal changes, including photoreceptor disruption and thinning of the outer nuclear layer, which limit the potential for functional recovery. In such switch populations, anatomical improvement does not necessarily translate into substantial visual gains, and stabilization of BCVA together with reduced treatment burden may still represent meaningful clinical benefit.

### 4.3. Considerations for Switch Populations

It should be noted that our study population consisted exclusively of switch cases, whereas the PHOTON trial enrolled treatment-naïve patients [6,7]. Switch populations may exhibit different disease characteristics, as these patients have typically shown suboptimal response to prior anti-VEGF therapy. The relatively low baseline CRT in our cohort (369.6 μm) compared to PHOTON (approximately 400 μm) likely reflects partial treatment effects from prior therapy. Additionally, the enhanced VEGF-binding capacity of aflibercept 8 mg may be particularly advantageous in eyes that demonstrate resistance to conventional-dose anti-VEGF agents [4,5]. While our findings suggest meaningful clinical benefit in switch cases, comparisons with PHOTON should be interpreted with caution given these fundamental population differences.

In interpreting our findings, it is also important to acknowledge that this study was designed as a single-arm retrospective pilot investigation without a control group. As historical data cannot serve as a valid comparator, the treatment effects observed here should not be interpreted as evidence of superiority over alternative regimens. The primary aim was to assess the feasibility, short-term efficacy, and safety of switching to aflibercept 8 mg using a PRN regimen in real-world practice.

### 4.4. Limitations

This study has several important limitations. Most notably, it was conducted as a single-arm retrospective pilot study without a control group, and historical data cannot serve as a valid comparator. Therefore, the magnitude of treatment effect should be interpreted with caution, and larger prospective studies with appropriate control groups are needed to validate these findings and determine the comparative effectiveness of aflibercept 8 mg under PRN dosing.

Second, the retrospective design and small sample size (20 eyes from 12 patients) limit statistical power and generalizability. Third, the heterogeneous treatment history and varying induction protocols introduce potential confounding. Fourth, the 6-month follow-up period is relatively short. Fifth, the PRN protocol was not standardized, and retreatment decisions relied on physician judgment. Finally, the observation that 25% of patients required only one injection could reflect selection bias related to inclusion criteria or clinician decision-making.

Although Bonferroni correction was applied for the primary outcomes, no imputation methods (including LOCF) were used, and all analyses were performed using observed data; therefore, the secondary analyses remain exploratory and should be interpreted with caution. Furthermore, due to limitations in model convergence with the current dataset, patient ID could not be incorporated as a random effect in the mixed-effects model, and inter-eye correlation arising from 20 eyes of 12 patients may not have been fully accounted for. The limited sample size and short follow-up period also restrict the strength of any safety conclusions.

## 5. Conclusions

Switching to aflibercept 8 mg using a PRN regimen reduced injection frequency while providing sustained anatomical improvement and short-term maintenance of visual function in eyes with diabetic macular edema. Although the numerical reduction in injection number was small (mean 1.45 injections over 6 months), this difference was statistically significant (*p* = 0.00231), and 70% of eyes achieved treatment intervals of ≥4 months, and 25% required only the switching dose. No intraocular inflammation or serious adverse events were observed. These real-world findings suggest that aflibercept 8 mg with PRN dosing represents a feasible and effective treatment approach for DME, offering a meaningful reduction in treatment burden while maintaining anatomical stability. Prospective studies are warranted to validate these observations and to compare PRN with alternative dosing strategies.

## Figures and Tables

**Table 1:** Patient Characteristics at Baseline.

Characteristics	Data
Number of patients/eyes, n/n	12/20
Age (years), mean ± SD	71.5 ± 14.0
Female, n (%)	5 (42)
Baseline BCVA (logMAR), mean ± SD	0.242 ± 0.252
Baseline CRT (μm), mean ± SD	369.6 ± 138.3
Previous aflibercept 2 mg, eyes (%)	17 (85)
Mean injection interval (6 months prior), days	81.16 ± 50.68
Mean number of injections (6 months prior)	1.55 ± 1.15
Types of Diabetic Retinopathy, n (%)	
-Mild NPDR	5 (25.0)
-Moderate NPDR	6 (30.0)
-Severe NPDR	4 (20.0)
-PDR	5 (25.0)

BCVA, best-corrected visual acuity; CRT, central retinal thickness; SD, standard deviation.

## Data Availability

The data are available from the corresponding author upon reasonable request.
